# Fall experiences of ambulatory children and adults with cerebral palsy: A qualitative study using thematic content analysis

**DOI:** 10.1111/dmcn.16474

**Published:** 2025-08-21

**Authors:** Marissa Esterley, Linda E. Krach, Kari Pederson, Sandy Callen Tierney, Nathan G. Wandersee, Elizabeth R. Boyer, The Cerebral Palsy Research Network

**Affiliations:** ^1^ Department of Research Gillette Children's St. Paul MN USA; ^2^ Department of Rehabilitation Medicine University of Minnesota Twin Cities Minneapolis MN USA; ^3^ Department of Orthopedic Surgery University of Minnesota Twin Cities Minneapolis MN USA; ^4^ Rehabilitation Science Graduate Program University of Minnesota Twin Cities Minneapolis MN USA

## Abstract

**Aim:**

To qualitatively assess the causes, adaptations, and psychosocial impact of falls, and solutions for safer environments, as shared by individuals diagnosed with cerebral palsy (CP).

**Method:**

Ambulatory adults with CP (*n* = 165; age median [interquartile range], range: 30 years [25–50], 18–76 years); 101 females, 59 males, five non‐binary/not specified) and caregivers of ambulatory children with CP (*n* = 151; age median [interquartile range], range: 10 years [7–14 years], 5–17 years; 64 females, 83 males, four non‐binary/not specified) responded to four open‐ended prompts regarding falls. Deductive and inductive thematic content analysis was conducted.

**Results:**

Eight themes were identified (psychological, physical, avoidance, adaptation, people, environment, policy, healthcare). Participants elaborated on the causes of falls (aging, physical, mental, environmental, situational), mechanics (most often trips), repercussions (psychological and physical), adaptations, difficulty getting up, and aspirations for themselves and society. Caregivers and adults detailed several adaptations to, or deliberate avoidance of, high‐risk situations (e.g. uneven surfaces, crowds). Specific suggestions for environmental accessibility (e.g. more handrails), societal behavioral responses (e.g. give autonomy, be patient), healthcare practice, and policy were made.

**Interpretation:**

This study offers deep insights into how individuals with CP navigate the challenges of falls and how people and surroundings both positively and negatively affect their fall‐related experiences. Many issues identified were multifactorial, requiring multidimensional, non‐ableist solutions. Participants were offered simple, but impactful, actions that could be taken immediately to support the creation of safer physical and psychological environments. More research and clinical practice guidelines are warranted.

## Abstract 2

**This original article is commented on by Nguyen on pages** 609‐610 **of this issue.**

Plain language summary: https://onlinelibrary.wiley.com/doi/10.1111/dmcn.70018

**What this paper adds**

- Fall‐related themes include psychological, physical, avoidance, adaptation, people, environment, policy, and healthcare.
- Adaptations to avoid falls were extremely common, demonstrating creativity and resilience.
- People should ask if and how to help when someone falls.
- Falls trigger anxiety, embarrassment, and avoidance, often outweighing the impact of physical injuries.
- Participation can be enhanced through safer and more inclusive activities and environments.

Ambulatory individuals with cerebral palsy (CP) often experience restricted mobility, increased unsteadiness, and elevated risk and concern for falling.^1^, ^2^, ^3^ Falls can cause embarrassment, activity avoidance, and injury.^3^, ^4^, ^5^, ^6^, ^7^, ^8^ Negative emotional and behavioral responses may become more prominent in adolescence,^8^ although high levels have been reported through middle adulthood.^3^ Over half of ambulatory children and adults with CP fall at least once per year,^3^, ^6^, ^9^, ^10^ which is two to three times higher than the general older adult population.^11^ Consequently, individuals with CP experience the physical and psychological sequelae of falls throughout their lives. Despite the higher risk of falls,^7^, ^12^ there is a dearth of literature on the lived experience related to falls.

Understanding CP and falls requires a multidimensional framework that considers the biological, psychological, and societal contributors to disability and health.^13^ One small study reflected this by identifying helpful and unhelpful attitudes, practices, and environment modifications that affect participation when confronted with the risk of falling.^8^ Avoiding activities and seeking support were common and sometimes beneficial,^5^, ^8^ but they can lead to isolation, reduced independence, and stigmatization, especially because of limited awareness of CP‐specific challenges by some healthcare professionals.^14^ Conversely, when healthcare professionals understand the daily lives of their patients, positive outcomes can occur.^14^ Increasing awareness of the lived experience surrounding falls will inform resource allocation, clinical services, and research. Hence, the purpose of our study was to describe the experiences of individuals with CP related to falls and their repercussions, and how clinicians and society can help mitigate the impact of falls across the lifespan.

## METHOD

The University of Minnesota Institutional Review Board (IRB) approved this study, which was part of a larger mixed‐method study of falls.^3^ Qualitative findings are reported in this article. The inclusion criteria were individuals with CP aged 5 to 89 years, classified in Gross Motor Function Classification System (GMFCS) levels I to III, who could read English. The IRB granted a waiver of written consent and assent. Instead, acknowledgment of consent was obtained from the caregivers of minors (< 18 years) and self for adults (≥ 18 years).

Recruitment occurred from January to October 2022 via e‐mails from individuals seen at Gillette Children's from 2011 to 2021, social media listings in 2022, and surveys e‐mailed to adults in the Cerebral Palsy Research Network Community Registry.

Research electronic data capture (REDCap, Vanderbilt University, Nashville, TN, USA) was used for screening, electronic consent, and survey administration. Caregivers were asked to consult their child, as appropriate. Four optional open‐ended prompts about falls based on the literature^8^ and conversations with adults with CP were created: (1) feel free to share any more information or provide clarifications regarding the above questionnaire on the consequences of falling; (2) describe how you may have adapted to perform certain activities to avoid falls; (3) provide clarifications regarding the above questionnaire on balance confidence and activity avoidance; and (4) provide any other comments about balance, trips, or falls, including what clinicians, families, or society can do differently to make physical and social environments safer or decrease your concerns about falls.

### Data analysis

Child, caregiver, and adult responses were analysed together. We used the framework method with deductive approaches guided by our research prompts and inductive approaches guided by thematic content analysis.^15^ Responses were analysed independently by two authors (ME, ERB), who began by reading all the responses. After familiarization, textual data were coded, and codes were organized into preliminary subthemes, maintaining alignment with the four open‐ended research prompts. Preliminary subthemes and a working codebook were created and continually refined as authors defined subthemes and associated central themes. A thematic framework matrix was created in an electronic spreadsheet where columns represented subthemes and rows represented participants. Throughout the charting phase, responses were mapped to identify recurring patterns and explore thematic relationships. People with lived experience (KP, SCT, NGW) informed the preliminary and final codebook and joined disagreement discussions to help reach consensus. After further review, subthemes were finalized to best represent the original responses.

## RESULTS

The final sample in the larger study included 381 participants (Table S1), of which 316 provided at least one response to the four optional open‐ended prompts (Table 1). Additional responses are found in Appendix S1.

**TABLE 1:** Demographics.

Characteristic	All, *n* = 316	Children, *n* = 151	Adults, *n* = 165
Age (years), median (25th centile, 75th centile) (minimum–maximum)	19 (10, 31) (5–76)	10 (7, 14) (5–17)	30 (25, 50) (18–76)
Sex			
Female	165 (52)	64 (44)	101 (63)
Male	142 (45)	83 (56)	59 (37)
Non‐binary/not specified	9 (3)	4 (3)	5 (3)
GMFCS level			
I	98 (31)	49 (32)	49 (30)
II	142 (45)	66 (44)	76 (46)
III	76 (24)	36 (24)	40 (24)
Topography			
Not specified	7 (2)	5 (3)	2 (1)
Unilateral (monoplegia)	20 (6)	11 (7)	9 (6)
Unilateral (hemiplegia)	94 (30)	53 (35)	41 (25)
Bilateral (diplegia)	97 (31)	37 (25)	60 (37)
Bilateral (triplegia)	30 (10)	21 (14)	9 (6)
Bilateral (quadriplegia)	64 (21)	23 (15)	41 (25)
Who answered the surveys			
Individual with cerebral palsy	138 (44)	2 (1)	136 (82)
Parent/primary caregiver	100 (32)	86 (57)	14 (8)
Parent/primary caregiver AND individual with cerebral palsy	74 (23)	60 (40)	14 (8)
Not specified	4 (1)	3 (2)	1 (1)

*Note*: Data are *n* (%) unless stated otherwise.

Abbreviation: GMFCS, Gross Motor Function Classification System.

Across all four prompts, two central themes—internal and external—and eight subthemes (internal: mental, physical, avoidance, adaptation; external: people, environment, policy, healthcare) emerged. Participants elaborated on the causes of falls, the mechanics, the repercussions, the frustrations, and the desired changes for themselves and society. Responses to the second prompt were divided into five additional subthemes related to avoidance (public, physical exertion, terrain) and adaptation (physical behaviors, mental). Internal themes are discussed first (Table 2) followed by external themes (Table 3).

**TABLE 2:** Example quotes from central theme 1 (internal) defined as thoughts, actions, or characteristics of the individual with CP.

**Mental**: mental ideas, thoughts, feelings, or emotions that an individual with CP can see as both the causes and effects or consequences of a fall
‘I am more concerned about the embarrassment of falling rather than the potential physical damage’. (Prompt 1) (Male, 29 years old, GMFCS level II, bilateral CP [triplegia])
‘… mental fatigue/being distracted is the cause of the overwhelming majority of my falls. If I'm not THINKING enough to pick up my foot, BAM’. (Prompt 1) (Female, 40 years old, GMFCS level II, bilateral CP [diplegia])
‘Sometimes I worry more about their reaction before even thinking about any pain. I worry people will see my falls and worry I shouldn't be independent. I worry they will stop seeing me as an equal and more as a liability’. (Prompt 1) (Female, 36 years old, GMFCS level II, bilateral CP [quadriplegia])
‘I have a lot of anxiety about falling. I've had panic attacks before and after falling. For years I refused to use an assistive device’. (Prompt 1) (Male, 49 years old, GMFCS level III, bilateral CP [quadriplegia])
‘I think fear and anxiety around falling contribute a lot to my inability to balance when performing some of the activities … The older I get, the less confident I am in my balance and the slower my reaction times become’. (Prompt 4) (Non‐binary, 35 years old, GMFCS level II, bilateral CP [diplegia])
**Physical**: the individual with CP's physical impairments that may cause a fall, or the physical/bodily consequences of falling
‘When [my child] falls it is often due to more than one variable. Example uneven surface and muscle fatigue. He often gets very angry and sad when he falls and lashes out’. (Prompt 1) (Caregiver of a 6‐year‐old male, GMFCS level II, bilateral CP [quadriplegia])
‘Often fall as a startle reflex’. (Prompt 1) (Male, 31 years old, GMFCS level I, bilateral CP [diplegia])
‘I wish it wasn't so hard for me to get up from the floor without assistance. That is why I am so fearful of falling’. (Prompt 1) (Female, 59 years old, GMFCS level III, bilateral CP [diplegia])
‘If I get nervous then my muscles tense up, and it takes a bit for them to calm down’. (Prompt 4) (Female, 32 years old, GMFCS level I, bilateral CP [quadriplegia])
‘If I am tired or in a hurry, I am more likely to fall’. (Prompt 4) (Female, 59 years old, GMFCS level II, bilateral CP [diplegia])
‘As a senior falls are more fearful now. That fear will also tighten the body up which increases the likelihood of falling. You worry more about injuries. As a younger person you got up quickly so no one would see you, shook it off and went on. Now if I fall I may need help to get up or have to crawl to some piece of furniture in order to get up. Decreases independence’. (Prompt 1) (Female, 59 years old, GMFCS level II, bilateral CP [diplegia])
**Avoidance**: activities or spaces intentionally not performed/attended by the individual with CP because of their real or perceived fall risk and/or any physical or mental repercussions if a fall occurs
‘It lowers confidence, increases fear and does not want to go out and access life as a result. Especially after a hard fall’. (Prompt 1) (Caregiver of a 14‐year‐old female, GMFCS level III, bilateral CP [quadriplegia])
‘I have fairly significant anxiety in large crowds as a direct result of all the falls I've experienced (and the subsequent fear of falling or being knocked over)’. (Prompt 2) (Female, 34 years old, GMFCS level III, bilateral CP [quadriplegia])
‘Sometimes I just sit there and watch everyone else have fun, because I hate my disease’. (Prompt 2) (Caregiver of a 16‐year‐old female, GMFCS level II, bilateral CP [diplegia])
‘[My child] will avoid sporting activities and other youth events because of the possibility he could fall. He's quit soccer because of this, dreads school track and field day, etc.’ (Prompt 2) (Caregiver of an 8‐year‐old male, GMFCS level II, bilateral CP [diplegia])
‘If there are a set of stairs/slope, I will absolutely find another route’. (Prompt 2) (Female, 30 years old, GMFCS level I, unilateral CP)
**Adaptation**: adaptations (physical, mental, or medical treatments) by individuals with CP because of their real or perceived fall risk and/or any physical or mental repercussions if a fall occurs
‘Fortunately I learned how to take a fall so that I can hopefully prevent major injuries’. (Prompt 1) (Male, 60 years old, GMFCS level II, unilateral CP)
‘Usually if he feels unbalanced, he will voluntarily fall, so the fall hurts less. I would say most of his falls are because he feels like the fall is coming, so instead of trying to catch or correct himself, he chooses to fall at his own pace’. (Prompt 1) (Caregiver of an 11‐year‐old male, GMFCS level II, bilateral CP [triplegia])
‘I oftentimes use my walker, brace myself against something or hold on to something while performing an activity where falling is a concern’. (Prompt 2) (Male, 26 years old, GMFCS level III, bilateral CP [quadriplegia])
‘I will psych myself up to do them. If I know it is something that I know there is no way I can do it, I will just automatically ask for help or not do it’. (Prompt 2) (Female, 32 years old, GMFCS level I, bilateral CP [quadriplegia])
‘I try to do risky things with optimal mental awareness and as slowly as possible in case I have to change my approach or adaptation midstream. Optimal mental acuity and muscle strength/tone is key for me; which is one reason I stay away from muscle relaxants. I live alone; so if I lose [sic] my ability to anticipate things and think things through in risky or potentially risky situations, I'm screwed. However, for me, now that I am older, most of the danger in falling is the difficulty/expenditure of energy and time in getting up off the floor’. (Prompt 2) (Female, 53 years old, GMFCS level III, bilateral CP [quadriplegia])

Abbreviations: CP, cerebral palsy; GMFCS, Gross Motor Function Classification System.

**TABLE 3:** Example quotes from central theme 2 (external) defined as thoughts, actions, or things external to the individual with CP.

**People**: the influence of individuals and society on the falls or consequences of falls for an individual with CP, including helpful or unhelpful influence or the need for change
‘It is also very difficult to fall in public because many bystanders want to get you on your feet immediately but I'm not always ready’. (Prompt 1) (Female, 54 years old, GMFCS level II, bilateral CP [diplegia])
‘Ask me if I need help getting up when I fall—and how I prefer to be assisted’. (Prompt 4) (Female, 64 years old, GMFCS level II, bilateral CP [diplegia])
‘Do not make us feel bad about falling. Let us be in control of how we feel safe in any given situation. Do not make us feel bad about the equipment we have. Do not treat us like a burden because we need equipment for an outing or more time’. (Prompt 4) (Female, 34 years old, GMFCS level II, bilateral CP [quadriplegia])
‘Please don't rush me … At least a quarter of my falls happen when I'm rushing myself or feel as if I am being rushed by someone and am trying to hurry to keep up with their expectations’.(Prompt 4) (Female, 34 years old, GMFCS level III, bilateral CP [quadriplegia])
‘Educate other children about CP so they continue to have an understanding of the condition and impact on children with CP … we have to find a way as kids expect [my child] to be as fast playing tag, sports, etc. They get frustrated and say challenging things to [my child] about being slow’. (Prompt 4) (Caregiver of an 8‐year‐old male, GMFCS level I, unilateral CP)
**Environment**: existing external indoor or outdoor (tangible) environment that influences the falls, or the consequences of falls, for an individual with CP, including helpful or unhelpful influences or the need for change
‘The slope issue is the absolute biggest problem I have … I can easily navigate downslopes of curb cuts into the street but my brain freezes me at upslope curb cuts. It's wild and infuriating!’ (Prompt 3) (Female, 40 years old, GMFCS level II, bilateral CP [diplegia])
‘My life would be easier if cities made sure sidewalks and roads are plowed and salted. I have taken time off work in winter to avoid risk of a fall due to ice or snow’. (Prompt 4) (Female, 24 years old, GMFCS level I, bilateral CP [diplegia])
‘Have better lighting, automatic doors, and level surfaces for entering eating establishments. Have the bathroom handicapped stall at the entry to the bathroom not in the back of the bathroom’. (Prompt 4) (Female, 59 years old, GMFCS level II, bilateral CP [diplegia])
**Policy**: governmental or institutional policies or law that influence the falls or consequences of falls for an individual with CP, including helpful or unhelpful influences or the need for change
‘The world is not made to accommodate people with special needs—regardless of the Americans with Disabilities Act’. (Prompt 1) (Female, 29 years old, GMFCS level I, unilateral CP)
‘Make parks, trails, stores, museums more accessible and fundraise for more ramps, handrails, and accessible events’. (Prompt 4) (Female, 33 years old, GMFCS level III, bilateral CP [diplegia])
**Healthcare**: healthcare systems or clinical care that influence the falls or consequences of falls for an individual with CP, including helpful or unhelpful influences or the need for change
‘I would love more training on how to fall safely or prevent falls …’ (Prompt 1) (Female, 54 years old, GMFCS level II, bilateral CP [diplegia])
‘Please stop providing patients with lifelong disabilities the same fall risk advice you provide elderly patients’. (Prompt 4) (Female, 28 years old, GMFCS level III, bilateral CP [quadriplegia])
‘PT has been helpful but doing something in a controlled context is different than being out in the community. It would be great if PTs could incorporate being outside into the sessions’. (Prompt 4) (Female, 28 years old, GMFCS level II, bilateral CP [diplegia])
‘As for clinicians, please validate my experience and help me find ways to do things I want to do’. (Prompt 4) (Female, 24 years old, GMFCS level I, bilateral CP [diplegia])

Abbreviations: CP, cerebral palsy; GMFCS, Gross Motor Function Classification System; PT, physiotherapist.

### Psychological

Multiple participants identified as ‘expert fallers’, stating that falls are a part of their lives and require acceptance, adaptation, and resilience. Psychological causes of falls reported were mental fatigue, distraction, and overthinking. Most responses centered on psychosocial repercussions, highlighting how perspectives like resilience or despondence can affect participation and quality of life. Participants struggled with embarrassment, anxiety, panic attacks, and insecurity; these affected their balance and were more worrying than the physical ramifications of falls. A caregiver mentioned that their child felt like other children noticed the physical difficulty of some tasks, increasing fear and insecurity. Others were resilient after falls. Caregivers wanted their children to feel empowered, not embarrassed, when asking for help.

### Physical

Participants described physical impairments contributing to falls, including fatigue, weakness, tight muscles, low tone, poor balance, and age‐related changes. Trips caused by foot clearance issues were the predominant cause of falls rather than slips. Some reported being startled, especially in public, as a substantial contributor to falls, prompting them to sometimes avoid public spaces. Children and adults alluded to excitement or nervousness causing muscle tension, which caused falls. Participants suspected comorbidities may also cause falls, like autism, seizures, cerebral or cortical visual impairment (visual impairment caused by damage to visual processing rather than eye structures), blindness, and cervical stenosis. The effects of falls included pain, minor injuries, and major injuries like fractures and concussion. Physical repercussions were reported to change over the lifespan, especially difficulty getting up.

### Avoidance

Participants described how concern about falling directly affected their participation. Many avoid crowds and public spaces. One participant, in middle adulthood, worried about falling and so avoids high‐risk situations because an injury could impede their ability to provide for their family as the primary household earner. Participants avoided uneven, irregular, slippery, or unknown terrains. One participant took time off work to avoid walking in wintry conditions. Others discussed taking longer routes to avoid precarious areas, staying on the perimeter of crowds and near walls for support. Avoidance of sports and physical activity was common for children and adults.

### Adaptations

Participants discussed physical and mental adaptations to mitigate fall risk, which were more common than avoidance examples (Table S2). Physical adaptations included sitting to dress and shower, crawling or scooting, wearing supportive shoes, strength and balance exercises, and using assistive devices, braces or orthoses, external structures (e.g. furniture, walls), or people for support. Scooting and crawling were reported by children and adults classified in GMFCS levels II and III. Some described using assistive devices (e.g. walker) to create space around them. Other physical adaptations included using hiking poles and looking down. Mental adaptations included hypervigilance and planning when going out in public. Participants noted the importance of concentrating on walking and paying attention to surfaces, objects, or crowds that might increase the risk of a fall. Other common themes included anticipation, understanding one's limitations, and learning when to seek help.

### People

Participants discussed how others helped or harmed their fall experiences. The presence or assistance of a companion for safety during daily activities (e.g. showering, especially reaching for items on the floor) was reported by many as helpful or necessary. Participants discussed frustrations and desires to change the reactions and behaviors of others; strangers can cause a fall or complicate recovery. People often felt crowded by strangers or rushed to move quickly. Asking people to remove shoes upon entering a house was shared as an example of ableism. Many experienced unwanted attention after falling in public. Participants urged strangers witnessing a fall to avoid overreacting; instead, they should ask if assistance is needed and then ask how they can help. A caregiver desired resources for their school to help educators understand, prevent, and respond to falls.

### Environment

The environment undeniably contributed to falls. Trip hazards included rugs, toys, uneven surfaces (e.g. curbs, stairs), and crowds. Familiarity affected fall risk and concern; stairs in one's home or school were not concerning, but stairs in unfamiliar spaces caused angst. Participants criticized misplaced or inadequate accessibility features. Many participants experienced anxiety on downward slopes, especially steeper slopes or those without handrails. Participants offered simple suggestions to improve accessibility, such as clearing public walkways (e.g. shopping aisles, sidewalks). While some wanted more slopes, others wanted fewer slopes, instead preferring low incline and deeper stairs. Decorations wrapped around handrails were criticized for obstructing use. One participant discussed the dangers of being outdoors in warmer climates because falls on the ground can cause burns, especially if it takes longer to regain an upright posture.

### Policy

Participants articulated that governmental or institutional policies can affect the incidence and consequences of falls, calling for enforcement of accessibility laws. Many advocated for better accessibility, primarily more ramps, elevators, handrails on each side of stairs and slopes, wider curb cuts, and accessible bathrooms. Participants advocated for more seating areas in schools, public spaces, and healthcare buildings. Residents in wintry areas desired prompt snow and ice removal and salting of walkways.

### Healthcare

A need for improvements in the healthcare system was expressed, emphasizing the importance of effective therapy, treatments, and adaptive equipment across the lifespan. A common request was teaching the correct falling techniques, particularly in childhood. Others said that they had been taught or naturally learned these techniques, such as rotating to minimize injury. Adults highlighted the lasting benefits of physical therapy for reducing fall risk, especially if it emulates real‐world spaces. They desire greater access to lifetime therapy. Some participants felt that healthcare professionals provided little or irrelevant fall information, especially for adults. Lastly, conducting therapy in realistic conditions was advocated for, including simulated curbs and slopes or having therapy in less controlled spaces. Treatments (e.g. botulinum neurotoxin A, selective dorsal rhizotomy, braces, orthopedic surgeries) were perceived by some to help and by others to worsen the incidence of falls. Individuals living in rural areas expressed the need for better adaptive equipment for rugged terrains.

## DISCUSSION

This study explored the causes and impacts of falls in individuals with CP across the lifespan, highlighting the complex interplay of several factors. Many acknowledged falls as a part of life, shaping how they navigate the world and managing their fall‐related concerns through different adaptation or avoidance methods to stay safe while finding self‐fulfillment. However, modifications to health‐related behaviors, the reactions and behaviors of others, inaccessible environments, healthcare practice, and policy must be addressed to systematically mitigate the risk of falling and associated repercussions.

Based on our findings, physical characteristics and comorbidities related to CP cause falls and change with age. Foot clearance is problematic. Many, including balance and fatigue, start to worsen in early or middle adulthood and are perceived to also cause functional decline.^4^, ^16^, ^17^, ^18^ Muscle tensing during emotional responses was reported and may represent dystonia; although dystonia is typically underdiagnosed,^19^, ^20^ it could increase the risk of a fall. Epilepsy and seizures are associated with increased risk of accidents and falls in the general population^21^ and were noted in this study. One participant attributed their child's fall propensity to cortical visual impairment. Like dystonia, cortical visual impairment is underdiagnosed but suspected to affect as many as 70% of this population.^22^ Studies are warranted to elucidate the patient characteristics with the greatest effect on fall risk and determine effective interventions.

The direct physical consequences of falls were omnipresent. The youthful ability to ‘bounce back’ after a fall may result in falls becoming insidious and ignored at clinical appointments in adulthood, when getting up becomes more challenging. Fractures are common^3^ and concerning because adults with CP face mortality and cardiovascular disparities after fractures^23^, ^24^ and are at a higher risk of prolonged recovery, affecting independence and quality of life. Adults experience fall consequences to a greater degree because of factors such as lower bone density, reduced muscle strength, and accelerated aging.^14^, ^25^ Individuals yearn for solutions to these problems,^18^ and participants recognized that exercise could be one solution. Empirical data on the type and incidence of fall‐related injuries, recovery, and preventive interventions is needed. Such information will help justify access to physical therapy or assistive devices to support mobility for individuals with CP because these factors were cited as barriers to more proactive clinical management.

Although fall‐related physical injuries have substantial implications, a key finding was that the emotional and psychological consequences of falls were both prevalent and potentially more detrimental to overall health than physical injuries. For the first time, fall‐related panic attacks in this population were documented. Worry or anxiety can manifest as poor balance,^26^ as can mental fatigue (from concentrating on the environment and not falling) or increased cognitive load.^27^ This, in turn, increases the risk of future falls. Some participants were resilient or unbothered by falls, but most voiced persistent concerns of anxiety, loss of confidence, loss of independence, and intentional activity avoidance, which aligns with previous studies.^3^, ^4^, ^5^, ^6^, ^7^, ^8^ Embarrassment was the most common emotion. Because embarrassment requires one to evaluate oneself against a social norm, alleviating it can be achieved by addressing the self‐evaluation or the social norm. Changing social norms and the acceptance of falls can especially help adolescents and adults who may feel falls are less socially acceptable compared to when they were children.^5^ The findings emphasize the critical importance of asking children and adults about the emotional impact of falls and the downstream effects on their life. Clinical tools like the Three Key Questions are available.^28^ Sometimes, referring the patient to a mental health professional may be necessary.

Understanding the physical and psychological impacts of falls is crucial because they can independently or collectively result in activity restriction from a young age, influencing participation in daily life (notably emphasized in the proposed updated description of CP)^29^ and shaping long‐term behaviors, place of residence, and career.^12^, ^18^ Individuals may decide to refrain from socializing.^5^, ^8^ This behavior can significantly affect mental health, particularly when paired with avoiding crowds or certain environments because of concern about falling or getting up. Such avoidance could indicate or lead to conditions like agoraphobia or posttraumatic stress symptoms, which are often underrecognized or underdiagnosed in individuals with CP.^30^ From a physical health perspective, persistent activity restriction (due to an injury or fear of falling) can reduce fitness and bone health, placing individuals at greater risk of serious injury and sedentary lifestyle‐related health conditions. Activity restriction or decreased participation may also result in cognitive issues, anxiety, and depression. The inevitable effects of aging magnify these factors, creating a vicious cycle of self‐perpetuating health challenges.

Insights from our study suggest that addressing the causes of falls and their consequences requires a multifaceted approach to create physically and psychologically safer environments. Proposed solutions combine individualized strategies, healthcare support, societal changes, and improved accessibility. Individualized strategies are largely mental, that is, resilience, adaptation, and pre‐planning. Others have documented similar risk management or adaptations to remain socially active.^5^, ^8^ Some strategies are learned while others may need to be taught.

Our data highlight the role of healthcare and clinicians in developing solutions, for which more research is warranted to inform evidence‐based practice guidelines on fall risk screening, assessment, and management specific to CP. Children may believe that they will outgrow falling, but it is a problem for all ages of ambulatory individuals.^3^, ^9^ Therefore, access to relevant fall‐related healthcare services and education across the lifespan is sorely needed.^12^, ^14^ Safe falling strategies should be taught at all ages; this requires therapist training and access to equipment to safeguard both the therapist and the patient. Furthermore, participants suggested that therapy in less controlled or familiar spaces, including crowds and public spaces, would improve generalizability to high‐risk fall situations, aligning with previous research emphasizing tailored fall prevention and the desire for healthcare professionals to understand daily challenges in CP care.^5^, ^8^, ^12^, ^14^, ^18^ Research is warranted to identify the prevalence and burden of falls, who is at the greatest risk, and which interventions and supports are most effective for prevention.

From a societal perspective, individuals felt that being physically and psychologically safer involves changing the environment and public behavior. To enhance participation, society needs to prioritize more inclusive activities and environments, especially at school during the formative years. Inappropriate or offensive public behavior was an impediment mentioned repeatedly and agrees with previous findings that onlookers affect self‐confidence and are a source of concern.^5^ Asking if and how to help, and showing greater patience and empathy, will solve many problems and reinforce individual autonomy.

Our findings suggest that addressing the shortcomings of public accessibility and policy needs to be part of the multifaceted solution. Misplaced or inaccessible features were common, leading people to feel unsafe and to restrict community engagement.^31^ Furthermore, unsafe or inaccessible environments can lead to absenteeism or individuals being forced out of work, resulting in significant economic and social impacts.^12^ While there were some conflicting perspectives for design specifics (e.g. more slopes vs more low incline stairs), it was clear that public accessibility needs to be enhanced. This can be achieved by including those with disabilities in the design of public spaces and a commitment from businesses to exceed the minimum accessibility requirements. Significant fear or anxiety around downslopes was highlighted in this study and elsewhere.^5^ Designing a shallower pitch with bilateral handrails will ensure that the space is wheelchair accessible and safer for assistive device users. Standards or laws are only as effective as their enforcement; greater enforcement of disability policies, regulations, and laws are merited. Collectively, all these supportive actions will foster a safe environment, mitigate the psychosocial distress of falls, and encourage participation rather than avoidance.^5^, ^8^

### Study limitations

Despite the novelty and strengths of this study, limitations remain. Participants may not represent the broader US or international population as they were predominantly invited via e‐mail from a US Midwest hospital. Participants were ambulatory, able to participate online, their own legal decision‐makers, with a maximum age of 76 years, and part of a larger fall study. Outspokenness or memorable experiences are probably overrepresented. Additionally, proxy reports from caregivers may inadequately represent their child's experience. Prospective studies could address potential recall bias.

In conclusion, this study provides an important contribution to the holistic causes of falls and how caregivers/children with CP and adults with CP experience the immediate and lasting physical and psychosocial effects of falls. These findings, coupled with quantitative studies, can inform a comprehensive fall risk stratification and assessment based on an individual's characteristics and behavior, the environment, and society's responses. Mitigating falls and their negative sequelae will require a coordinated, multidisciplinary effort.

## Supporting information


**Table S1:** Number of eligible participants by stage.

**Table S2:** Number of comments pertaining to subthemes that emerged from the four optional open‐ended prompts.

**Appendix S1:** Additional responses for the four open‐ended prompts.

## Data Availability

The data that support the findings of this study are available from the corresponding author upon reasonable request.
