# Supplementary material for: Fall experiences of ambulatory children and adults with cerebral palsy: A qualitative study using thematic content analysis
Source: Dev Med Child Neurol. 2025 Aug 21;68(5):673–80. doi: 10.1111/dmcn.16474 (PMC13056018; doi:10.1111/dmcn.16474)
Supplement: Supplementary file 1 — Table S1: Number of eligible participants by stage. [file DMCN-68-673-s003.docx]

**Table S1.** Number of eligible participants by stage.

| **Stage** | **N** | **Reasons for exclusion/not advance to next stage** |
| --- | --- | --- |
| Potentially eligible participants invited | 2,505 | invalid email, didn't complete screening, not interested |
| Participants who completed screening to confirm eligibility | 1031 | didn't meet all inclusion criteria/screen fail |
| Eligible participants who consented to participate | 728 | didn't complete consent, completed wrong consent, not interested, not interested in this specific study (CPRN adults), didn't answer at least 1 outcome survey |
| Consented participants who answered at least 1 outcome survey for the broader falls study | 381 | Did not provide an optional answer for at least 1 of the 4 open-ended prompts for qualitative data analysis |
| **Participants in the qualitative data analysis** | **316** | *Breakdown by Prompt* |
|  | 131 | Prompt 1 (clarification regarding consequences of falling) |
|  | 308 | Prompt 2 (adapt to avoid falls) |
|  | 91 | Prompt 3 (clarification on concern about balance and activity avoidance questionnaire) |
|  | 130 | Prompt 4 (additional comments, change external factors for safety) |
