# Supplementary material for: Fall experiences of ambulatory children and adults with cerebral palsy: A qualitative study using thematic content analysis
Source: Dev Med Child Neurol. 2025 Aug 21;68(5):673–80. doi: 10.1111/dmcn.16474 (PMC13056018; doi:10.1111/dmcn.16474)
Supplement: Supplementary file 2 — Table S2: Number of comments pertaining to subthemes that emerged from the four optional open‐ended prompts. [file DMCN-68-673-s002.docx]

**Table S2.** Number of comments pertaining to subthemes that emerged from the four optional open-ended prompts.

| **8 Subthemes** | **Examples** | **Prompt** | | | **Avoid/Adapt Subthemes** | **Prompt** |
| --- | --- | --- | --- | --- | --- | --- |
|  |  | P1. Share any more information or provide clarifications regarding the above questionnaire on consequences of falling. | P3. Provide clarifications regarding the above questionnaire [on concern about balance and activity avoidance] | P4. Comments about balance, trips, or falls, including what clinicians, families, society can do differently to make physical & social environments safer or decrease your concerns about falls. |  | P2. Describe how you may have adapted to perform certain activities to avoid falls. |
| **Internal:** Psychological | Embarrassment, fear, mental fatigue, determination | 86 | 26 | 25 | **Avoid:** Public | 6 |
| **Internal:** Physical | Impairments, aging, injury | 123 | 48 | 38 | **Avoid:** Physical Exertion | 55 |
| **Internal:** Avoid | Avoid people, terrain, activities | 3 | 26 | 5 | **Avoid:** Terrain | 24 |
| **Internal:** Adapt | Adapt physically, psychologically | 45 | 59 | 36 | **Adapt:** Physical Behaviors | 502 |
|  | | | | | **Adapt:** Psychological | 106 |
| **External:** People | Caregivers, society | 40 | 28 | 70 |  | |
| **External:** Environment | Terrain, home adaptation | 23 | 21 | 121 |  |  |
| **External:** Policy | Enforcement, change | 6 | 4 | 111 |  |  |
| **External:** Healthcare | Use (services, products), CP-specific | 26 | 6 | 35 |  |  |
