# Supplementary material for: Fall experiences of ambulatory children and adults with cerebral palsy: A qualitative study using thematic content analysis
Source: Dev Med Child Neurol. 2025 Aug 21;68(5):673–80. doi: 10.1111/dmcn.16474 (PMC13056018; doi:10.1111/dmcn.16474)
Supplement: Supplementary file 3 — Appendix S1: Additional responses for the four open‐ended prompts. [file DMCN-68-673-s001.docx]

**Appendix S1**. Additional responses for the four open-ended prompts.

| **Prompt 1: Share any more information or provide clarifications regarding the above questionnaire on consequences of falling.** |
| --- |
| Falling is part of having cerebral palsy but the severity of my injuries grew as I reached middle age and my muscles naturally shortened as well. I've always fallen often but I got by with mostly bruises and scrapes until after age 35…People also assume I'm not intelligent because of my falls, yet I have a high IQ. However, the most difficult part for me of falling is the loss of confidence in my physical abilities…. *[54y Female, GMFCS II, Diplegia]* |
| Some of my falls result because of my foot (on the leg with an AFO) not rising high enough. Other times, it's because this foot gets caught on something. *[Female 41y, GMFCS II, Hemiplegia]* |
| I have had so many falls that I can't remember them all. I only really recall the major ones, and even that is difficult as there are too many. All end up at least with major bruising, pain. No fracture - yet - but I have osteopenia, so very aware that this time might come. *[Female 56y, GMFCS I, Diplegia]* |
| He says why can't he be like a normal kid and not fall *[Caregiver of 5y Male, GMFCS II, Diplegia]* |
| Very mild CP, most of my falls occurred when I became a runner, especially with longer distances (4+ miles) when I couldn't lift my right foot up high enough and it dragged on the ground. *[Male 27y, GMFCS I, Hemiplegia]* |
| I have always experienced strong psychological distress when I fall in public, however I feel less alone since I have had my mobility assistance dog. He is always with to help me stand up and support me emotionally afterwards! *[24y, GMFCS III, Diplegia]* |
| I've noticed since turning 40 that my body doesn't bounce back like it did when I was younger. A fall now results in lengthy hospital stays. I've had 2 temporary admissions (6 weeks total) to two different care facilities for convalescence and rehab, until staff deemed I was safe to return home. *[Female 54y, GMFCS III, Triplegia]* |
| It depends if I am wearing my brace if I fall or not. *[Male 21y, GMFCS I, Hemiplegia]* |
| When I fall in public I really do feel like people look down on me and laugh at me and I really do wish I didn't fall as much *[Male 18y, GMFCS II, Diplegia]* |
| I don't like it when strangers rush to my aid when I fall. They used to do so, but since the pandemic that is less frequent and I'm thankful for it. It causes more anxiety to have people surrounding me and then I don't feel like I have enough room to gain my balance once getting back up. *[Female 29y, GMFCS II, Diplegia]* |
| In addition to my spastic diplegia cerebral palsy I am also legally blind. I am not sure how much this contributes to the falls. *[Male 21y, GMFCS II, Diplegia]* |
| I do not like the attention if I fall. I don't like it when people react to me as if I can't do things *[Male 25y, GMFCS II, Diplegia]* |
| …she has definitely fallen more than20 times this past year. It seems that when he is tired or fatigued her chances increase more! *[Caregiver of 13y Female, GMFCS II, Triplegia]* |
| I often fall forward onto my hands and knees resulting in frequent scrapes to my knees. I have also broken my knee cap twice. *[Non-binary 33y, GMFCS II, Hemiplegia]* |
| Falls most often occur when trying to walk independently and he gets going too fast. *[Caregiver of 13y Male, GMFCS III, Triplegia]* |
| It is never a good feeling to fall. When I was younger, I would get up really fast and usually be able to brush it off and keep moving. For the most part, that is what I still do. However, I have noticed that as I am getting older, it is harder to just bounced back and keep going. It is getting harder to get up without something hang on to. I worry more about falling than I ever have before. I intentionally make choices to avoid falls…I also sometimes feel angry that I have to deal with this in the first place. I have also noticed that when wearing certain shoes I am more prone to falling. Sometimes it makes me angry that I cannot just wear cute shoes. it sounds silly, but it can be frustrating. I have sent very cute shoes to the Goodwill after falling. I have two toddlers, and they leave toys all over the floor at my house. I am constantly picking up toys and trying to keep my path clear. I feel like since I had kids, I do fall more because there are more obstacles in my path…. *[40y Female, GMFCS II, Diplegia]* |
| My son prefers to crawl and climb to get around at home. He sometimes falls while standing at counters/tables at home, almost daily, but does not always get hurt. He mostly falls when he walks with his Walker/crutches, and mostly uses shoes and braces to walk. He often falls to his knees but doesn't always get hurt. He gets hurt most often from climbing, especially at playgrounds and parks. *[Caregiver of 7y Male, GMFCS III, Quadriplegia]* |
| I don't really fall. I used to trip but it is better now *[15y Female, GMFCS I, Monoplegia]* |
| Says sorry a lot when falls and/or injures self *[Caregiver of 8y Female, GMFCS II, Triplegia]* |
| She doesn't fall that often, but when she does, she does feel embarrassed about it and wishes she was like the other kids. *[Caregiver of 10y Female, GMFCS III, Triplegia]* |
| My son can be very unstable when walking. It is hard for him to keep up with his peers. He gets easily frustrated and tends to give up (embarrassed). *[Caregiver of 5y Male, GMFCS I, Hemiplegia]* |
| When [NAME] falls it is often due to more than one variable. Example uneven surface and muscle fatigue. He often gets very angry and sad when he falls and lashes out. *[Caregiver of 6y Male, GMFCS II, Quadriplegia]* |
| He gets frustrated when he falls. He uses a walker and is trying to carry items or play while standing and he loses his balance quite a bit. He gets walking quickly and his feet get tangled up and he falls. *[Caregiver of 8y Male, GMFCS III, Triplegia]* |
| …She falls at least once daily by her legs randomly giving out. She falls on her bottom but is able to catch her fall with her leg muscles and goes down slowly so she doesn't get hurt *[Caregiver of 6y Female, GMFCS III, Hemiplegia]* |
| The older he gets now he blushes *[Caregiver of 17y Male, GMFCS II, Triplegia]* |
| The number of total falls since age 4 is a very rough estimate (5-10 per year), but it's clear that I've been falling my entire life. I have had periods of time relatively free of falls, and others where I've fallen more frequently. *[43y Female, GMFCS II, Hemiplegia]* |
| I have never broken a bone in a bone in a fall. I have a lot of anxiety about falling. I've had panic attacks before and after falling. For years I refused to use an assistive device. Over the past 7 years, I've been helped a lot by a sports psychologist. We count walking as my sport. I am now at peace with using hiking poles. I want to get the message out about the importance of mental health health in any way I can. *[49y Male, GMFCS III, Quadriplegia]* |
| I lot my falls cause me a lot of pain. I often worry about pain that will not end and will have lasting affects. I also worry my falling will put me in situations that I do not want to be in or will cause me harm in my future. I do not like the amount of scrapes, bruises and other problems falling causes. When I fall a lot of people will blame me and ask what the problem is or assume my condition is a lot worse than it is. *[34y Female, GMFCS II, Quadriplegia]* |
| I often feel bad for scaring people. Sometimes I Worry more about their reaction before even thinking about any pain. *[36y Female, GMFCS II, Quadriplegia]* |
| I seem to not care what people think any more, seeing me fall…. *[65y Male, GMFCS III, Hemiplegia]* |
| Just walking differently from others makes me vulnerable to preconceived thoughts that my disability goes beyond physical. It does not. So add a fall to the mix and it really sets the tone of being even more incapable! Frustrating!! *[53y Female, GMFCS II, Quadriplegia]* |
| Getting up can be easy depending on severity of the fall. Minor falls I can get up quickly. *[23y Female, GMFCS I, Hemiplegia]* |
| I wish it wasnt so hard for me to get up from the floor without assistance. That is why i am so fearful of falling *[59y Female, GMFCS III, Diplegia]* |
| Don't fall that much amazingly for one with CP. I'm also legally blind which contributes to the falls when they do happen (not seeing the change in terrain (uneven ground) to pick up my feet etc) fast enough to avoid the fall…. *[60y Male, GMFCS I, Hemiplegia]* |
| No issues of falling as a young adult, just when she was a toddler/preschool *[22y Female, GMFCS I, Diplegia]* |
| For me the embarrassment would come from the fact that I am able to pass as nondisabled, but a sudden fall would likely lead to having to disclose my disability & a potential change in perception by others. *[30y Female, GMFCS I, Hemiplegia]* |
| I think the people around me end up being more concerned about my fall then I actually am *[28y Female, GMFCS II, Diplegia]* |
| Most of my falls were caused by foot drop (which caused tripping and falling) in addition to falls caused by poor balance. I do trip often which can lead to more falls. *[30y Female, GMFCS II, Hemiplegia]* |
| She never wears socks alone. *[Caregiver of 5y Female, GMFCS III, Diplegia]* |
| I dont fall very often but I wish it never happened at all I'm grateful for the abilities I do have compared to others that have cp *[31y Female, GMFCS II, Monoplegia]* |
| Many falls are caused by balance issues due to spasticity. Many occur during transitions (e.g. going from sitting in chair to standing in walker). *[23y Male, GMFCS III, Quadriplegia]* |
| I fall less after my last surgery, and if I wear my brace. *[12y Male, GMFCS I, Hemiplegia]* |
| [NAME] is a very determined little girl. I don't think she would be as far as she is if she didn't get right back up (refusing help) and keep moving forward after each fall *[Caregiver of 7y Female, GMFCS III, Triplegia]* |
| I don't fall often since I do try to be very careful while walking in a walking device. *[Caregiver of 6y Male, GMFCS III, Diplegia]* |
| [NAME] is generally fearless and falls frequently. She typically gets right up and continues on. Sometimes she does not indicate she is injured until we see a bruise or scrape. Numbers and percentages are guesstimates as we do not track these events nor do we make a big deal over them, we encourage her to brush off these incidents and not to let them discourage her from continuing in her activities. She also likes to climb so some of her falls are as a result of losing her grip in her weak arm such as on climbing apparatus' , slides or trees, at playgrounds in the lake or whereever. *[Caregiver of 7y Female, GMFCS II, Hemiplegia]* |
| In the summer time here is Arizona falls outside are specially daangerous because the ground is so hot and burns. It takes time for me to get up or for someone to help me get up. *[Caregiver of 12y, GMFCS III]* |
| She falls a lot, for her it's just part of life. Fortunately/unfortunately it doesn't really cause her to make different choices. She tried a Zumba class this spring, she did a kids fun run, she fell, gets patched up and goes back to it. *[Caregiver of 7y Female, GMFCS III, Hemiplegia]* |
| [NAME] falls many times every day. No mater what we do or what she wears falling happens a lot. Most of the time there is little we can do to predict or prevent them. *[Caregiver of 5y Female, GMFCS III, Diplegia]* |
| Feel more embarrassed when people think they need to help. *[34y Male, GMFCS II, Quadriplegia]* |
| I just had SDR as an adult 8 months ago so my falls have been fewer than prior to surgery, but it's now harder to get up. Also, since the pandemic I have been less active in my community and working from home, so less commuting means less falling. *[28y Female, GMFCS II, Diplegia]* |
| The world is not made to accommodate people with special needs - regardless of the Americans with Disabilities Act. Add in cold weather elements and getting around outside of your home is very difficult if you are unsteady on your feet. Unless you have a special need, you don't understand this. Also, if you rent your home, this can be a challenge. *[29y Female, GMFCS I, Hemiplegia]* |
| Someone is usually with an arm's length of our son during times of mobility because we are terrified of falls. That's why he has had so few. Our Hypervigilance has gotten much worse since spinal fusion in 2018 *[22y Male, GMFCS II, Quadriplegia]* |
| His muscle tone gives out and he clasps backward a lot. He can not stand up in a gait trainer for more than 1/2 hour and then he loses the strength and ability to support himself even in a gait trainer or with someone while walking. Picking him up can be hard sometimes when he can't seem to get his feet underneath him and pop up with assistance. It varies on a given day how his muschle tone will do. *[19y Male, GMFCS III]* |
| …I caught my foot on the carpet and it kind of rolled up with me. A man coming in behind me tried to help me up, but at the time I banged my knee pretty good, so I just had to sit there for a minute for the pain to subside before I could stand. I think that is the worst part of falling in public. People always try to come help you, but sometimes it's just easier to use a wall or something else inanimate after taking a minute to recover from the pain/embarassment. *[33y Male, GMFCS II, Quadriplegia]* |
| She feels this way when falling around people outside of her immediate family. When she falls around her family she does not feel embarrassed. *[Caregiver of 7y Female, GMFCS II, Diplegia]* |
| I feel like his shoe lift is a big culprit cause it drags and catches on the ground. *[Caregiver of 7y Female, GMFCS I, Hemiplegia]* |
| My son is self motivated and has the passion and desire to walk. Even when he fall, he will get up by himself and will continue his training. However, he gets upset a few time when people around. This give me the impression that he does not like others to see him fall *[Caregiver of 10y Male, GMFCS III, Quadriplegia]* |
| He is very confident despite falling a lot. *[Caregiver of 8y Male, GMFCS II, Triplegia]* |
| Depends on if falls occur at home, work, or public place. Falls at work are most difficult. *[56y Male, GMFCS I, Hemiplegia]* |
| …Sometimes falling factors are a combination of several things (muscle fatigue + uneven surface) so I didn't know how to account for those. I think you should also differentiate between falls caused by tripping and falls without tripping. *[27y Female, GMFCS II, Triplegia]* |
| I trip frequently. Usually at least once a day. Most of the time it does not result in a full fall, but does result in loss of balance *[28y Female, GMFCS I, Monoplegia]* |
| … Because my right side is slightly stronger, I have a tendency to fall to the left and damage that side of my body more. *[40y Female, GMFCS II, Diplegia]* |
| As I get older my falls seem to result in more permanent injury (can't wear my wedding ring on my ring finger because I've done something to the joint) *[48y Female, GMFCS I, Hemiplegia]* |
| As I am aging I fall much less in motion and more likely from balance issues getting up from the couch in the middle of the night when I have been sleeping and my muscles have less tone in them *[53y Female, GMFCS III, Quadriplegia]* |
| I no longer carry groceries to my vehicle, I always use a cart in order to avoid falling or tripping. *[54y Female, GMFCS II, Diplegia]* |
| At 54, I have no time for embarrassment. I'm an expert Faller🤣 *[54y Male, GMFCS II, Diplegia]* |
| At times I may be walking fine and then all of the sudden I get nervous and feel as if I am going to fall and then will walk very slowly and be very tight. I may not be in pain right away it maybe that evening or the next few days…. *[59y Female, GMFCS II, Diplegia]* |
| As a senior falls are more fearful now. That fear will also tightens the body up which increases the likelihood of falling. You Worry more about injuries. As a younger person you got up quickly so no one would see you, shook it off and went on. Now if I fall I may need help to get up or have to crawl to some piece of furniture in order to get up. Decreases independence. *[59y Female, GMFCS II, Diplegia]* |
| …the only time I fell with crutches was after surgery and I couldn't lift the crutches over the cracks in the sidewalk. I don't have broken bones pre sé but 4 fractures. Two in my spine and I fractured my 8th rib twice. I also fell in the bathroom once too hard and wound up fracturing all 8 fingers. I have arthritis in my SI joint because I keep falling on it but more on the left side. It's very hard for me to use any type of walking aid because I need to have my good hand free when walking. It's either I walk or I succumb to a wheelchair. I refuse to do that until absolutely necessary. *[59y Female, GMFCS I, Hemiplegia]* |
| Serious fall was result of undiagnosed cervical stenosis. *[61y Female, GMFCS I, Diplegia]* |
| My first major fall was at 2yrs and resulted in my breaking my nose and a concussion. After that, I feel daily until I was given ice skating lessons …As an adult in recent years, my falls started up again due to the vision problems and my right leg collapsing. Major traumatic injuries resulted and I had to have surgeries (and or wear casts) after each of them. I'm now frequently receiving NeuroPT where we work on managing all aspects, recognizing that the CP is also lurking with the CVI. *[61y Female, GMFCS II, Quadriplegia]* |
| I have had several recent knee surgeries for replacement and revision due to infections as well as a quad tendon repair. At age 60 and with the quick succession of procedures rehab has been very difficult and prolonged. Four years ago I was at my high water mark of 2.5 miles walking on uneven terrain with no need to stop. That is the goal I want to get to hopefully reducing number of future falls. *[61y Male, GMFCS I, Hemiplegia]* |
| There's times when I fall I feel like I'm a board I'm stiff when I go down. Or if when I'm falling I try to turn around and land on my butt. *[62y Female, GMFCS II, Diplegia]* |
| since my car accident, and resulting foot/leg fractures, I can no longer get up off the floor by myself *[69y Female, GMFCS III, Triplegia]* |
| Anymore, because it is so difficult to get up and because of the changes a fall can create regarding my condition, I cannot afford to fall. *[70y Male, GMFCS III, Diplegia]* |
| I am still ambulatory but increasingly use a walker (or shopping cart) to assist my mobility. I have led a very active lifestyle including [competitive sports]. I have never had really good balance, but in recent years my balance has definitely deteriorated which contributes to more falls or risks thereof. AND, the fear of falling clearly impacts my choice of activities. About 10 years ago I stopped using my regular stock 2-wheeled bicycle in favor of a high grade recumbent trike. *[76y Male, GMFCS II, Quadriplegia]* |

AFO: ankle-foot orthosis, CVI: Cerebral (or cortical) Visual Impairment, NeuroPT: neurologic physical therapy, SDR: selective dorsal rhizotomy

| **Prompt 2: Describe how you may have adapted to perform certain activities to avoid falls.** |
| --- |
| I always have my partner home when I take a shower in case of in case of slips and falls. I never left anything over my head and frequently ask for assistance if something out of my reach. I don't currently engage in sports of any kind. And I usually have someone else accompanying me during outings. *[29y Female, GMFCS II, Diplegia]* |
| …Turn and rotate as I fall to avoid injury if possible. *[13y Female, GMFCS II, Triplegia]* |
| …I avoid stairs if I can and use lifts if they are available. Although, if the stairs have a rail, I can use them slowly. Reaching for stuff above my head is ok. Reaching for stuff on the floor is sometimes hard, so I have a grab stick that I use sometimes. Walking up slopes is ok. Walking down can be problematic because of my balance, so I generally either ask for help or avoid it.…. *[40y Female, GMFCS II, Diplegia]* |
| I moved to an apartment with an elevator, and a shower for a person with disabilities, and a washer and dryer in the apartment. *[62y Female, GMFCS II, Diplegia]* |
| I have to use a walker at school to keep kids from bumping me and making me fall. Even though I can walk well with a stick and hate using the walker. *[12y, GMFCS III]* |
| I sit down while getting dressed. Sometimes when my muscles are stiff I shower sitting down. *[27y Female, GMFCS II, Quadriplegia]* |
| Going with people I trust, having a plan, wearing my braces and sleeping enough before event *[23y Female, GMFCS I, Hemiplegia]* |
| I walk slower or concentrate on keeping my feet straight so I don't trip because they turn in so much *[41y Female, GMFCS I, Diplegia]* |
| More time spent in a wheel chair *[19y Male, GMFCS III, Quadriplegia]* |
| When getting dressed I sit on the floor *[33y Female, GMFCS III, Triplegia]* |
| …sliding down somewhat risky slopes on my butt *[26y Female, GMFCS I, Hemiplegia]* |
| He walks slower, holds on to the railing or side of wall, needs help carrying items inside if he's going upstairs in the house *[Caregiver of 7y Male, GMFCS II, Diplegia]* |
| He holds on to surfaces and/or people to ensure that he maintains his balance or flat out refuses to do it. *[Caregiver of 7y Male, GMFCS II, Hemiplegia]* |
| … Not participating in sports where I could fall *[9y Female, GMFCS II, Diplegia]* |
| … use forearm crutches only when the ground is smooth and fairly even… *[14y Female, GMFCS III, Diplegia]* |
| Hold railings, don't walk in stocking feet on hardwood *[Caregiver of 6y Male, GMFCS I, Hemiplegia]* |
| I made up a song for during hiking to watch for roots and rocks… *[14y Female, GMFCS I, Quadriplegia]* |
| … he has gotten really good at falling safely. He has developed an instinctual ability to protect his head as he falls. *[Caregiver of 8y Male, GMFCS I, Diplegia]* |
| Strength training *[17y Male, GMFCS I, Quadriplegia]* |
| Choose activities that I know my ability can handle *[13y Male, GMFCS I, Hemiplegia]* |
| I have also started using a hiking stick while walking around outside (uneven ground and long walks). *[41y Female, GMFCS II, Hemiplegia]* |
| Careful stepping on stairs and elevators, slow steps on slopes (especially while walking down), gripping onto handrails for stairs, and hanging onto walls when ice skating *[27y Female, GMFCS I, Diplegia]* |
| I prefer special Olympics. I do not ski or skate due to my brain injury. I ride a 3 wheel bike. I also continue to work on strength and balance at exercise medicine *[18y Male, GMFCS II, Diplegia]* |
| … have my assistance dog help me with tasks like picking up clothing items, so that I don't have to bend over and risk a fall…. *[24y, GMFCS III, Diplegia]* |
| When ice skating this past winter, I used some broom ball shoes that were spongey on the bottom, so they gripped the ice better…. *[24y Male, GMFCS I, Hemiplegia]* |
| … I also use rolling suitcases to carry things to avoid falls. I look for accessible routes for hikes and take Ubers instead of walking…. *[33y Female, GMFCS III, Diplegia]* |
| I make sure I have a good wide base with my feet before reaching for things above my head… *[21y Male, GMFCS II, Diplegia]* |
| My cerebral palsy only affects my left hand and wrist, so if I fall it's not a result of my CP, but I do adapt by trying to protect that wrist and hand if I do fall. *[25y Female, GMFCS I, Monoplegia]* |
| … Always assisted with steps, walking in grass and steep slopes. We always do a bath, showers too risky for falls…. *[Caregiver of 5y Male, GMFCS III, Diplegia]* |
| Wear proper shoes, move slowly, know limitations. *[13y Female, GMFCS I, Hemiplegia]* |
| When I got Botox it helped *[10y Female, GMFCS I, Hemiplegia]* |
| [NAME] often chooses not to participate or he asks his dad or myself to carry him. *[Caregiver of 6y Male, GMFCS II, Quadriplegia]* |
| Uses wheelchair and is carried. *[Caregiver of 6y Female, GMFCS III, Hemiplegia]* |
| She relies on assistance from people to navigate more challenging terrain like stairs, slopes, uneven ground or unfamiliar territory. She refuses to use other types of assistance such as a walker or cane (sensory issue). *[Caregiver of 6y Female, GMFCS III, Quadriplegia]* |
| Grab bar on trampoline. Adaptive bike used outdoors daily *[Caregiver of 7y Female, GMFCS III, Diplegia]* |
| I crawl. *[9y Female, GMFCS III, Triplegia]* |
| Assess surroundings ahead of time, practice, bring a chair, bath chair, stay behind, use wheelchair in crowds, use shopping carts as support, electric carts, recumbent bike, walk near wall, use rails, support person on uneven surface, take frequent breaks. *[Caregiver of 13y Male, GMFCS II, Triplegia]* |
| …hold a hand or use a wall or put hands on stairs if there is no railing or scoot on butt. For Hockey he plays sled hockey. *[Caregiver of 7y Male, GMFCS I, Triplegia]* |
| …In winter I use walking poles and crampons for my boots. *[24y Female, GMFCS I, Diplegia]* |
| We have a lot of conversations about taking our time and not running. Running increases the chance of falling much more for him. We wear prism glasses now which his significantly increased his ability to see more clearly all around him and he has less falls because of it. *[Caregiver of 6y Male, GMFCS I, Hemiplegia]* |
| I currently use custom orthotics and trekking poles as much as I can…. *[43y Female, GMFCS II, Hemiplegia]* |
| …I have an epic family and friend support team that look out for me. They will help make accommodations when needed. *[53y Female, GMFCS II, Quadriplegia]* |
| Furniture walking,,,use a cane and use a walker for walking longer distances. *[59y Female, GMFCS III, Diplegia]* |
| …I have my 'wing people' (friends that can tell me when terrain changes) that can alert me to possible problems (stairs in restaurants, hidden slopes etc.) *[also legally blind]* *[60y Male, GMFCS II, Hemiplegia]* |
| … People are typically understanding and super willing to help if I speak up. *[40y Female, GMFCS II, Diplegia]* |
| If there are a set of stairs/slope, I will absolutely find another route. Or, if it is an activity, I will find reasons to avoid it. *[30y Female, GMFCS I, Hemiplegia]* |
| …My family has added additional rails to staircases to assist with safety as well…. *[30y Female, GMFCS II, Hemiplegia]* |
| When standing up from a sitting position, I pause to collect my balance. *[25y Male, GMFCS II, Diplegia]* |
| I like to try things like skating but don't try for very long. Some of my difficulties are a result of balance and poor vision. *[7y Female, GMFCS II, Hemiplegia]* |
| Playing activities that are adaptable to CP - Archery, four wheeler, art, goalie. *[Caregiver of 6y Male, GMFCS II, Hemiplegia]* |
| …Use AFOs. Make them feel like they are part of her just like shoes so that it's not something that makes her different. *[Caregiver of 7y Female, GMFCS III, Hemiplegia]* |
| I live in a city and will often Uber or Lyft if there is a lot of street walking involved. I prefer transferring trains to get me closer to my destination rather than walking on the street. *[28y Female, GMFCS II, Diplegia]* |
| Use assistance of PCA. (Or, cognitive delay minimizes understanding of risk.) *[30y Male, GMFCS II, Hemiplegia]* |
| Our son also has nonverbal autism and is prone to running and has an unsteady gate while walking as it is. When he starts to dart away we shout "walk" if he gets away. In public, we hold his elbow while walking in case he starts to dart away, or to be fast to steady him if he stumbles while walking *[2y Male, GMFCS I, Quadriplegia]* |
| If I'm in a crowded place I do my best to stay by the walls whenever possible or generally on the outskirts of people even if it takes me a little longer to get to where I want to go. Even in none crowded areas I kind of gravitate towards the wall, so I have something to brace against if I need it. *[33y Male, GMFCS II, Quadriplegia]* |
| re: getting dressed, i sit down to put my clothes on. over the last ~3 years (thanks, pandemic & aging!), raising my arms above my head has put me way more off balance than it used to. i purchased a long grabber to access high items in my kitchen and got a laundry cart to bring my laundry to & from the laundry room in my building because there's a slope to get into it. i got grab bars in my shower six years ago and that's reduced my fall fear SIGNIFICANTLY. i do experience leg buckling when i'm nervous or standing for a period of time (undefined - sometimes short, sometimes 15-20 mins and i can never figure out how long the time period will be). but i haven't yet fallen because of that. *[40y Female, GMFCS II, Diplegia]* |
| … Where headphones if I'm worried that a loud noise would s to startle me and I would fall…. *[13y Female, GMFCS III, Diplegia]* |
| …I will use my AFOs or Bioness (FES device) to keep from dragging my toes and tripping…. *[27y Female, GMFCS II, Triplegia]* |
| …Wearing shoes that fit well and are appropriate for the activity *[28y Female, GMFCS I, Monoplegia]* |
| …and i wear appropriate footwear. I wasn't always the best about that as a kid. *[39y Female, GMFCS I, Hemiplegia]* |
| I use my rollator walker more often outside and in some instances when I haven't had enough sleep the night before (for example)… *[46y Female, GMFCS II, Diplegia]* |
| stopped wearing flip-flops. try to stretch more often. do balance exercises *[52y Female, GMFCS I, Monoplegia]* |
| I put one hand on the wall of the shower when rinsing my hair because closing my eyes makes me fall. I had a pneumatic lift placed on my bicycle seat so that I can put my feet to the ground before stopping my bike ( instead of leaning to the side). I wear flats with an ankle strap or sneakers almost all of the time. *[54y Female, GMFCS II, Hemiplegia]* |
| Taking my time Deep breathing and "talking to myself" to relax… *[56y Female, GMFCS I, Diplegia]* |
| Learn to do reaching/grasps one handed so the other hand can steady the body. *[59y Female, GMFCS II, Diplegia]* |
| … I rely heavily on proprioception and my leg braces to manage and catch myself before I fall. The braces help me keep my right leg from collapsing--that helps reduce that huge risk… *[61y Female, GMFCS II, Quadriplegia]* |
| mom has piggybacked me so we could hike through the redwood forest. I walked some with my sticks. *[5y Female, GMFCS III, Diplegia]* |
| When in a store shopping, I will use a shopping cart or a scoter when available. *[76y Male, GMFCS II, Quadriplegia]* |

AFO: ankle-foot orthosis, FES: functional electrical stimulation, PCA: personal care assistant

| **Prompt 3: Provide clarifications regarding the above questionnaire** *[on concern about balance and activity avoidance].* |
| --- |
| Post SDR my biggest challenge is numbness. I still can't feel the bottom of my right foot, so I haven't done anything physically intense because I'm afraid of loosing my balance. *[28y Female, GMFCS II, Diplegia]* |
| I had a hip replacement last year, which lengthened that leg to evenly match the other one. I'm still getting used to noticing balance changes, my depth perception has changed, and my anxiety is greater in social outdoor settings. *[46y Female, GMFCS II, Diplegia]* |
| I think my balance is getting much worse with age :( *[39y Female, GMFCS I, Hemiplegia]* |
| Due to my recent circumstances of my walking getting worse, I have to avoid many activities all together. *[30y Female, GMFCS II, Monoplegia]* |
| I trip very often, I catch myself before I actually fall to the ground/floor. *[14y Female, GMFCS I, Quadriplegia]* |
| I still do the activities but I use a wheelchair to do so. Without the wheelchair I'd avoid most public activities. *[34y Male, GMFCS II, Quadriplegia]* |
| Does not avoid activities that cause falling, unless he has just fallen and still hurts. Is willing to do/try anything physical. He does hold onto railing for stairs and we have taught him "sit to get down" when he is unsteady. He only does this because of repetitive instruction not out of fear. *[Caregiver of 6y Male, GMFCS I, Hemiplegia]* |
| Social event like Skating/skiing/sledding – [NAME] would require alot of holding and carrying. These activities would be high risk of falling and getting hurt so we do not do these types of activities *[Caregiver of 7y Female, GMFCS II, Diplegia]* |
| …being vigilant about uneven surfaces; removing trip hazards at home and places we vacation *[54y Female, GMFCS II, Diplegia]* |
| There are many activities which present a high risk of falls, but other aspects of my condition, such as limited physical ability, make it difficult for me to perform those activities anyway *[23y Male, GMFCS III, Quadriplegia]* |
| I walk independently w/o crutches. So I must be well balanced when I walk, before I walk. *[25y Male, GMFCS II, Diplegia]* |
| It's not always "I don't currently do this activity" ....much of our sons CP doesn't allow some of the activity just because his abilities. Falling may not be why he can't do it. *[Caregiver of 13y Male, GMFCS III, Quadriplegia]* |
| [NAME] has adapted really well to life in an AFO right leg brace for walking. He had leg lengthening surgery in 2018 or 2019. His falling immediately went down with elimination of tip-toe walking. *[Caregiver of 8y Male, GMFCS I, Hemiplegia]* |
| Once I am familiar with stairs then I am not as concerned. Example - i am comfortable on the stairs at my house and school but not at a new spot like a theatre or sports complex. *[15y Male, GMFCS II, Quadriplegia]* |
| I have a high fear of falling so crawling is the most safe way for me to achieve any task. *[Caregiver of 9y Female, GMFCS III, Triplegia]* |
| Falls are always a concern, and happen randomly. While it is always possible, he doesn't let the fear of falling stop him from doing things. *[Caregiver of 8y Male, GMFCS II, Diplegia]* |
| … we have anti slip bath mats all over the bathroom floor to prevent slipping… *[22y Male, GMFCS II, Quadriplegia]* |
| I never played formal sports but I ran around sometimes with other kids. My feet would get tangled up and I would trip. So I don't run very much. I tried a scooter and rollerskating and always fell. I have a three-wheel bike that I like to ride. I also like to ride horses. *[29y Female, GMFCS I, Hemiplegia]* |
| Reaching above my head doesn't throw me off balance, but reaching down for me is really difficult from a standing position and I generally avoid it unless there is something I can hold on to. *[33y Male, GMFCS II, Quadriplegia]* |
| My mom always by myside to support me. Also, I understand that crawling is safer for me. *[10y Male, GMFCS III, Quadriplegia]* |
| … Getting in or out of a chair- The office chairs where I work have wheels which make them more challenging to get in and out of. At times when I find myself at a point where I am getting in and out of the chair a lot to help customers, I will simply push the chair in the corner and continue to stand until the situation quiets down…. *[35y Male, GMFCS II, Hemiplegia]* |
| I'm not scared to fall at school because I know there are people there that will help me. *[Caregiver of13y Female, GMFCS III, Diplegia]* |
| All numbers are estimates. I don't usually keep track of how often I fall, since it is a frequent occurrence. *[27y Female, GMFCS II, Triplegia]* |
| I will not climb stairs without railing even while using mobility aids as I am afraid of falling and the anxiety makes it even harder to balance. Walking up inclines is not usually a problem for me but going down steep inclines does trigger anxiety and again makes it harder to balance. *[35y, GMFCS II, Diplegia]* |
| Just have my first fall where I needed to get emergency care. Usually when I fall I only scrape my knees/hands. I think my falls are caused by foot drop. *[52y Female, GMFCS I, Monoplegia]* |
| To be clear, while balance is a huge issue for going up and down stairs, the real issue is my knees will not do it (they were wrecked during a series of surgeries from age 13-18). *[54y Female, GMFCS III, Triplegia]* |
| I regularly do not lift my feet high enough, so I often trip on stairs or uneven surfaces. I now use a four footed cane to help me stay balanced. I worry about tripping over my in turned feet. *[62y Female, GMFCS II, Diplegia]* |
| My balance is what is most affected in regard to my CP. I can not run 10 feet without falling but I can walk for miles.I would be described as a left hemi, but my poor balance is causing me more challenges than anything else. I do no have spasticity, but more so posturing in flexion and some lacking end range of motion. *[54y Female, GMFCS II, Hemiplegia]* |
| In order for me to stop falling as much, I have to concentrate really hard. Because if I start thinking of something else I fall. Sometimes my feet crisscross in front of each other then I fell. Like I did the other day. *[62y Female, GMFCS II, Diplegia]* |
| When I have too much to keep track of while in motion, and there is too much visual input to process; if also there is too much auditory noise, or too much crowding--i am more likely to trip and fall than when in a quiet and low key familiar place. Cerebral Visual Impairments can go with balance/ataxia issues and there is something known as a CVI meltdown where the brain just can't handle it all and shuts down. Most of my falls had this mix of problems behind them. *[61y Female, GMFCS II, Quadriplegia]* |
| Though I have a concern about falling, there are certain activities (e.g., showering) that I must do. *[70y Male, GMFCS III, Diplegia]* |
| Over the years I have become more adept at grabbing a close by solid object when I am falling....wall, railing, piece of furniture, tree, car, etc. This process most often prevents my falling to the ground, but it doe NOT preclude some degree of injury most often to my upper torso, arm/hands and head. When incurred, these injuries have included cuts and abrasions, soft tissue bruising and even mild concussions. *[76y Male, GMFCS II, Quadriplegia]* |

AFO: ankle-foot orthosis, CVI: Cerebral (or cortical) Visual Impairment, SDR: selective dorsal rhizotomy

| **Prompt 4: Provide any other comments about balance, trips, or falls, including what clinicians, families, society can do differently to make physical & social environments safer or decrease your concerns about falls.** |
| --- |
| Most of what comes to mind has to do with large-scale changes at a societal, cultural, and communal level. AFFORDABLE ACCESSIBLE HOUSING is a HUGE issue; it would be an ENORMOUS help to live in an environment adapted to ME instead of having to adapt my body and balance to where I live. (Thereby putting myself at risk.) MANY more handrails and access buttons on the entrances of ALL public buildings; businesses actually being compelled to update and modify their locations so that they are in line with ADA. (Yes, EVEN IF THE BUILDING IS OLD AND IT MIGHT REQUIRE ADDITIONAL FUNDING OR INCONVENIENCE.) Properly made cutouts that don't compel you to walk an extra fifteen feet just to get to them. CONSISTENTLY salting and clearing sidewalks or parking lots in the winter. Handicap-accessible stalls that are actually wide enough to take a walker in with me. On a more personal level, good old-fashioned patience and empathy is rarer and more precious than I have words for. PLEASE don't rush me. Don't glare at me if I need an extra minute; the world won't stop spinning. Don't shame me for doing what I need to do to be safe. Don't assume I need your help if I'm moving a little more slowly on a given day - but don't assume I don't want it, either. ASK ME WHAT I NEED TO GET FROM ONE POINT TO ANOTHER SAFELY, EFFICIENTLY, AND WITH DIGNITY. In other words, treat me like a person first, NOT a task you have to get through as quickly as possible so you can get on with the rest of your day. At least a quarter of my falls happen when I'm rushing myself or feel as if I am being rushed BY someone and am trying to hurry to keep up with their expectations. *[34y Female, GMFCS III, Quadriplegia]* |
| Where does one begin.........there is a real lack of information/research about the impact of aging on pre-existing disability and the value and type of exercise. Our current health/insurance system limits the amount/length of PT services which I have found to be very beneficial. Although we have made significant public policy advancements, societal and, yes, family attitudes continue to marginalize and place low expectations on persons with disabilities. The COVID pandemic has starkly illustrated the degree to which we as a nation devalue persons with disabilities and our aging population. Programs and services designed to benefit those of us with disabilities have been severely curtailed and the lost benefits may never be recovered. Perhaps folks like myself with a secure income, good education and a sophisticated support network will persevere, but these assets including good health care and insurance DO NOT offset my daily concerns relative to the increased likely- hood that a serious fall may soon have a very negative impact on my daily living. *[76y Male, GMFCS II, Quadriplegia]* |
| I need more regular physical therapy. I fall less when I have PT at least 2x a week. Balance training really helps. *[12y, GMFCS III]* |
| Move to a warmer state during the winter. *[56y Male, GMFCS I, Hemiplegia]* |
| Even on my three-wheel bike, if I don't hit the sidewalk opening just right, I will tip over. The sidewalk opening is just barely wide enough for my wheels. *[29y Female, GMFCS I, Hemiplegia]* |
| it should be acceptable for people to use handrails on either side of stairs. it should be taught that it is ableist to have people remove shoes when entering your house. *[45y Male, GMFCS I, Diplegia]* |
| Have railing especially on outside steps where there are no walls to lean on… *[64y Female, GMFCS II, Diplegia]* |
| Design less areas with slopes, and try not to make stairs as steep (shallower stairs are easier to prevent the fear of falling)…. *[27y Female, GMFCS I, Diplegia]* |
| More ramps and handicap parking would be helpful when we're out and about. Stores and other buildings could do a better job at clearing the walkway - sometimes displays take over the majority of the aisle and it's hard to pass through. *[19y Male, GMFCS III, Quadriplegia]* |
| Sport complexes (hockey/football arena's, etc) have seating available with minimal or no steps that isn't designated for "wheel chairs". We have been to places that we end up sitting on metal folding chairs in the wheelchair space. *[Caregiver of 15y Male, GMFCS II, Quadriplegia]* |
| When I go on family trips where I have to walk and move around a whole lot my legs and muscles become weak and then I worry about falling *[18y Male, GMFCS II, Diplegia]* |
| Fix uneven sidewalks more regularly, replace street curbs with curb ramps and install handrails on sloped sidewalks *[30y Female, GMFCS II, Hemiiplegia]* |
| His balance has improved since SDR. He can stand for short periods of time but tips over very easily. He tries to take steps but tips right away. With any type of excitement he tenses up with things so that doesn't help things. I feel like he will get excited and then stiffen up and then looses balance and falls. *[Caregiver of 5y Male, GMFCS III, Diplegia]* |
| A lot of my falls are just down to my knees and I just get up again and keep going. Social environments could be safer if there were more ramps and less stairs and a handrail to hold on to. *[13y Female, GMFCS III, Diplegia]* |
| A big risk factor is the pace society moves at. *[Caregiver of 8y Female, GMFCS II, Diplegia]* |
| Epilepsy sometimes leads to fall also *[Caregiver of 5y Female, GMFCS II, Monoplegia]* |
| If everyone would just slow down, I could stay caught up. This goes for my body muscles and my thinking brain - everything goes too fast and I think people lose patience with me very quickly *[14y Male, GMFCS II, Hemiplegia]* |
| Some clinics (not Gillette) still do not have auto opening handicap doors. In public, a general patience for those who walk more slowly/carefully would be greatly appreciated. *[Caregiver of 6y Female, GMFCS III, Quadriplegia]* |
| Needs 1:1 support for safety *[Caregiver of 7y Female, GMFCS III, Diplegia]* |
| I'm embarrassed when I trip or fall and I get mad when someone tries to comfort me or make me feel better *[Caregiver of 13y Male, GMFCS II]* |
| Design AFO's as footwear, hard plastic with metal hinges or pieces is extremely painful, results in days of healing, fixes and modifications resulting in loss of solid footing. resources (understanding of falls and what to do) for school, assistance for proper distance walking, between class support, sitting in school , making supplies available. Curbs with access grip/bar, big stores have benches throughout store or quick seat chair *[Caregiver of 13y Male, GMFCS II, Triplegia]* |
| I think schools should have ramps between levels vs only have stair options. I think there needs to be more education about kids with CP and how it impacts what they can do while at school to keep up with other kids. *[Caregiver of 11y Male, GMFCS II, Hemiplegia]* |
| We'd love a better assistive device for ice skating. *[Caregiver of 11y Female, GMFCS I, Hemiplegia]* |
| Although the questionnaire focuses on falls, I have almost daily "tripping" episodes where I catch my toe on nothing and have to stumble to catch myself. I have run into door frames, desks, coffee tables etc because of this. I *hate* the concerned gasp and "oh my god are you ok?" Response I often get from people who witness my fall or trip. I will tell you if I'm not ok. Just let me get on with my day without treating me like a small child…. *[24y Female, GMFCS I, Diplegia]* |
| … Post signs when there is slippery floors - he falls a lot when it's slippery. Adaptive parks so he has the ability to play safely. *[Caregiver of 6y Male, GMFCS I, Hemiplegia]* |
| Having people walk close behind me is stressful due to my concerns about balance and falls. I also don't like to be rushed…. *[43y Female, GMFCS II, Hemiplegia]* |
| …Just before Covid hit, I took a vacation to Florida. I'm sure the shower was up to code. There were grab bars and a shower chair. Unfortunately, the grab bars were in the wrong spots. They were pretty much worthless to me, even if I moved the shower chair…. *[49y Male, GMFCS III, Quadriplegia]* |
| I wish there was a way to engineer my own walker. A DPT at Gillette ([NAME]) and I tried to find one that would work in my circumstances, but none were safer than a Guardian *[56y Male, GMFCS II, Quadriplegia]* |
| Better lighting….Try to avoid creating environments where I have shift directions quickly *[36y Female, GMFCS II, Quadriplegia]* |
| Tough one-remove the stigma...just because you are physically impaired doesn't mean you can't think for yourself….*[53y Female, GMFCS II, Quadriplegia]* |
| …One other thing-don't rush me! That makes my body go into spasms mode & that throws everything off. *[53y Female, GMFCS II, Quadriplegia]* |
| Winter is the worst, I truly feel like I cannot go out ,since most stores, resturants do not keep sidewalks or parking lots salted. *[59y Female, GMFCS III, Diplegia]* |
| …the number of times I've seen no accessible access is insane *[28y Female, GMFCS II, Diplegia]* |
| I'm half way through my 60th year and have fortunately not had any serious injuries with falls & such. my balance seems to be fine yet. I am at that stage in life, however, that I am taking things a bit slower than I used to and have a little less dare devil attitude about hills, slopes stairs and uneven ground. *[also legally blind] [60y Male, GMFCS II, Hemiplegia]* |
| I actually think the patient needs to be encouraged to ask for help and not feel embarrassed in that regard. *[26y Male, GMFCS I, Diplegia]* |
| …In general, an increase in awareness is helpful. People that don't struggle with this don't even realize that their is no railing going into their house. I look at their house and I see I huge obstacle to overcome, they may not even see the problem *[40y Female, GMFCS II, Diplegia]* |
| USUALLY DON'T WEAR SHOES OR FLIP FLOPS DUE TO CONCERN OF LOOSING RIGHT SHOE AND MAKING IT HARDER TO WALK & ESPECIALLY RUN. *[18y Female, GMFCS I, Hemiplegia]* |
| Educate other children about CP so they continue to have an understanding of the condition and impact on children with CP. Difficult to teach or communicate to other children at young ages. However, we have to find a way as kids expect [NAME] to be as fast playing tag, sports, etc. They get frustrated and say challenging things to [NAME] about being slow. [NAME] starts to believe he cannot participate in tag or soccer, and the list may continue into 2nd grade. *[Caregiver of 8y Male, GMFCS I, Hemiplegia]* |
| Some ramps seem too steep *[Caregiver of 13y Male, GMFCS III, Quadriplegia]* |
| Strangely the best floor we've come across has been the pool at swimming lessons. It is roughed concrete and she can use her crutches even when it is wet. *[Caregiver of 5y Female, GMFCS III, Diplegia]* |
| Clearly mark uneven ground with yellow paint. *[Caregiver of 9y Female, GMFCS I, Hemiplegia]* |
| Don't overreact to falls. Nothing is worse than having those around her 'panic react' when she just wants to get on with her life. Stop training people to treat her that she is so different, she's just a kid being a kid, sometimes they fall. *[Caregiver of 7y Female, GMFCS III, Hemiplegia]* |
| It would be nice to have a informational card the size of a business card to hand to a person leading an activity or sports or social activity to alert them of possible issues with balance or muscle fatigue without having to feel put on the spot if the situation comes up. *[Caregiver of 10y Female, GMFCS I, Hemiplegia]* |
| Because I have poor balance walking is difficult and since we live on a farm rough surfaces are even more difficult to walk on. I need better adaptive equipment to hold up to the rough terrain on a farm. *[Caregiver of 9y Female, GMFCS III, Triplegia]* |
| In a perfect world people would be more aware of others around them and give them space and help when needed. *[Caregiver of 8y Male, GMFCS II, Diplegia]* |
| … We plan ahead for what we're doing and take the wheelchair if we're going to need it. Caroline's Cart at Target and Cub are amazing because we can leave the wheelchair at home. I wish more shopping places like Walmart had them *[22y Male, GMFCS II, Quadriplegia]* |
| Our concerns have more to do with epilepsy, but there are also balance issues, possible vision issues, that also contribute. He will say at times, "I'm shaking or I'm falling." I'm not sure if this is C.P. related as he has had shaking or if underlying seizure activity. Either way, it is clear in those moments he feels unstable… *[30y Male, GMFCS II, Hemiplegia]* |
| Strategic placement of hand holds and avoid particularly hard or sharp materials in corners. My only two serious falls in my lifetime involved metal doorframes or corners…. *[28y Female, GMFCS III, Quadriplegia]* |
| There was only one single room apartment in my city for rent, which met my physical disabilities and I am not yet in a wheelchair! *[62y Female, GMFCS II, Diplegia]* |
| Considering accessibility is a big issue in major cities. There's often tons of construction and a lack of curb cuts…. *[28y Female, GMFCS II, Diplegia]* |
| It would be nice if building whether professional buildings or places to gather such as churches, community centers would be required to have a wheelchair accessible entrance so he could enjoy life more and make it easier to be mobile. *[19y Male, GMFCS III]* |
| Falls backwards were most concerning before having SDR surgery. Since the surgery I can regain balance easier and falls backwards have significantly decreased. I mostly fall forward now. Having things to hold on to or slowing down the speed of activities is most helpful. *[Caregiver of 8y Male, GMFCS II, Triplegia]* |
| More elevators would help. Safer stairs. I would like more places to sit down when needed. *[17y Male, GMFCS II, Monoplegia]* |
| …I feel grateful for the friends and family in my life who offer a supportive arm or shoulder when I need it without being patronizing. *[35y, GMFCS II, Diplegia]* |
| To avoid tripping I don't do anything strenuous. *[30y Female, GMFCS II, Monoplegia]* |
| … Mandatory wider doorways and corridors would help those of us in wheelchairs…. *[40y Female, GMFCS II, Diplegia]* |
| …More postings about that assistance is available for mobility impaired people as they provide at airports. We needs that everywhere. *[43y Female, GMFCS II, Diplegia]* |
| …The type of pavement slabs used in city centre are very slippy even in nice weather so maybe think about using different ones *[49y Female, GMFCS I, Diplegia]* |
| I wish more stores had benches to sit on when shopping and I avoid restaurants that only have high barstool type chairs as they are extremely hard for me to get in and out of..also, many hospitals and dr offices have long distances to walk in order to receive services and benches along the hallways would be helpful. I don't use a wheelchair and don't want to. Little stops along the way would be a great help! *[54y Female, GMFCS II, Diplegia]* |
| I have had poor balance since I was born at 25 weeks. It has gradually gotten worse and is now almost non-existent. Standing in one place holding onto my walker is the hardest thing I can do, I have to keep moving to keep my balance. The sidewalks and entrances into buildings in Saskatchewan Canada are in really rough shape and very uneven. Most entrances have steps making it impossible for me to enter. Bathrooms tend to be closet size, no room for equipment or grab bars of any kind. *[54y Female, GMFCS III, Triplegia]* |
| The Wilderness Battlefield and Spotsylvania had a composite trail through some areas that was possibly (shredded rubber?) That was easier to walk on ...and it leveled the ground too. *[54y Male, GMFCS II, Diplegia]* |
| Provide more elevators near escalators. I actually have pretty good balance for walking but vanity gets the best of me and I try to wear shoes that I shouldn't. (heels or lack of ankle strap) *[54y Female, GMFCS II, Hemiplegia]* |
| Allow for pt/ot throughout one's lifespan *[56y Female, GMFCS II, Quadriplegia]* |
| Even footpath No 'subtle' changes of levels or tripping hazard (uneven pavement, unmarked changes of level, especially if small) Handrails, grabrails everywhere there is a change of level, i.e. steps or slopes A 'fall log' might be very useful to log in every single fall, its circumstances and consequences. That might help to recall, but also measure the frequency and severity and spot any changes in patterns. Based on my experience with migraine log which helped identified triggers. *[56y Female, GMFCS I, Diplegia]* |
| Mandate that old buildings, regardless of age or design, that are used by the public be made handicapped accessible per the ADA immediately or closed. *[60y Male, GMFCS II, Diplegia]* |
| Have better lighting, automatic doors, and level surfaces for entering eating establishments. Have the bathroom handicapped stall at the entry to the bathroom not in the back of the bathroom. Make more of an effort to wipe up spills that happen on public floors. *[59y Female, GMFCS II, Diplegia]* |
| Lift assist oven rack so that you can get hot/heavy things out of the oven one handed. *[59y Female, GMFCS II, Diplegia]* |
| Losing my balance is the reason for most of my falls. I wish that society could understand that accessibility means more than just having a ramp at the main door, or safety rails around a toilet. I need to be able to open a door while using a walker or a scooter. *[61y Male, GMFCS I]* |
| Many of my falls are usually related to extreme activities. *[61y Male, GMFCS I, Hemiplegia]* |
| have Medicare allow wheelchairs for the longer and more likely to fall trips outside the home; *[67y Male, GMFCS I, Diplegia]* |
| In order for me to stop falling as much, I have to concentrate really hard. Because if I start thinking of something else I fall. Sometimes my feet crisscross in front of each other then I fell. Like I did the other day. *[62y Female, GMFCS II, Diplegia]* |
| I don't want to be a burden to anyone *[69y Female, GMFCS III, Triplegia]* |
| I wish designers would seek more input from the disabled with regard to the design of buildings, entrances, sidewalks, etc. rather than simply designing such structures from their assumptions regarding the needs of the disabled community. *[70y Male, GMFCS III, Diplegia]* |
| A reaction is everything. When people react calmly i'm able to hop right back up and keep going. If they gasp and gush over me asking if im ok etc then its embarrasing. *[Caregiver of 6y Male, GMFCS II, Hemiplegia]* |
| Burn all rugs forever. Rugs are evil pieces of trash. :) *[38y Female, GMFCS III, Quadriplegia]* |

ADA: Americans with Disabilities Act, AFO: ankle-foot orthosis, COVID: Coronavirus Disease 2019, DPT: Doctor of Physical Therapy, ot/pt: occupational therapy/physical therapy, SDR: selective dorsal rhizotomy
